# Evaluation of the effectiveness of environmental disinfection by no touch hydrogen peroxide technology against MDR bacteria contamination and comparison with active chlorine disinfectant

**DOI:** 10.1186/2047-2994-4-S1-P43

**Published:** 2015-06-16

**Authors:** M Ferrari, A Bocconi, A Anesi

**Affiliations:** 1 City Hospital Lodi, Lodi, Italy

## Introduction

The multi-resistant organisms (MDRO) survive for long periods of time in a variety of surfaces in hospital environments with high risk of infection trasmission.

## Objectives

The objective of this study was to evaluate effectiveness and non-inferiority of a disinfection system based on H2O2 and Ag+ micro-mist, vs. chlorine procedure, by monitoring the reduction of microbial contamination on room surfaces.

## Methods

Active chlorine (5.000 ppm), vs. decontamination system based on a solution of 5-8% H2O2 and 60 ppm active Ag+ (1mL/m^3^ intensity of treatment) were compared. Two beds 26 rooms located in different wards mainly within the Departments of Medicine and Rehabilitation were previously occupied by patients infected by MDRO. Environment and medical equipment disinfection procedures were performed prior to a new bed occupancy in addition to routine cleaning activities. 10 surfaces were sampled in the hospital room. Microbial colonisation was assessed at Time 0 (T0) before cleaning, T1 immediately after cleaning and T2 after disinfection procedures, using swabs on a surface area of approximately 57 cm^2^. All swabs were inoculated with standard procedure and evaluated on CFU per cm^2^. Organisms were identified by standard microbiological methods.

## Results

780 surface samples were collected: 600 from rooms treated with H2O2, 180 with active chlorine. Before cleaning the surfaces, all samples collected in the rooms resulted colonised, with an average density of mesophile organisms up to 56 CFU/57 cm2 (range 0-400). MDROs were isolated from samples collected in 20/26 rooms respectively. After manual cleaning with detergent and active chlorine disinfection, an average density of organisms of 15 CFU/57 cm2 (range 0-270) was recorded. MDROs were found from samples collected in 2 rooms but only after an enrichment step. After H2O2 disinfection, a density of bacteria in the range of 0 and 3 CFU/57 cm^2^ was observed and no MDROs were found.

## Conclusion

Our data indicate that the hydrogen peroxide and active silver ions disinfection system, together with manual cleaning procedures, is non inferior vs. active chlorine based procedure. Hydrogen peroxide resulted effective in minimizing the overall microbial load on the hospital room surfaces and in eradicating MDRO.

## Disclosure of interest

None declared.

